# Correlation Between Smoking Paradox and Heart Rhythm Outcomes in Patients With Coronary Artery Disease Receiving Percutaneous Coronary Intervention

**DOI:** 10.3389/fcvm.2022.803650

**Published:** 2022-02-11

**Authors:** Han-Ping Wu, Sheng-Ling Jan, Shih-Lin Chang, Chia-Chen Huang, Mao-Jen Lin

**Affiliations:** ^1^Department of Pediatric Emergency Medicine, China Medical University Children's Hospital, China Medical University, Taichung, Taiwan; ^2^Department of Medicine, College of Medicine, China Medical University, Taichung, Taiwan; ^3^Department of Medical Research, China Medical University Children's Hospital, China Medical University, Taichung, Taiwan; ^4^Department of Pediatrics, Children's Medical Center, Taichung Veterans General Hospital, Taichung, Taiwan; ^5^School of Medicine, National Yang-Ming Chiao-Tung University, Taipei, Taiwan; ^6^School of Medicine, Kaohsiung Medical University, Kaohsiung, Taiwan; ^7^Department of Cardiology, Taipei Veterans General Hospital, Taipei, Taiwan; ^8^Department of Public Health, Chung Shan Medical University, Taichung, Taiwan; ^9^Department of Medicine, Taichung Tzu Chi Hospital, The Buddhist Tzu Chi Medical Foundation, Taichung, Taiwan; ^10^Department of Medicine, College of Medicine, Tzu Chi University, Hualien, Taiwan

**Keywords:** percutaneous coronary intervention, smoker's paradox, stable coronary artery disease, risk factors, long-term outcome

## Abstract

**Background:**

The effect of smoking on short-term outcomes among patients with acute coronary syndrome (ACS) undergoing percutaneous coronary intervention (PCI) is controversial. However, little is known about the impact of smoking on long-term outcomes in patients with stable coronary artery disease (CAD) who receive PCI.

**Methods:**

A total of 2,044 patients with stable CAD undergoing PCI were evaluated. They were divided into two groups according to smoking status (current smokers vs. non-smokers). Baseline characteristics, exposed risk factors, angiographic findings, and interventional strategies were assessed to compare the long-term clinical outcomes between groups. Predictors for myocardial infarction (MI), all-cause death, cardiovascular (CV) death, and repeated PCI procedures were also analyzed.

**Results:**

Compared with non-smokers, current smokers were younger and mostly male (both *P* < 0.01). They also had a lower prevalence of chronic kidney disease (CKD) and diabetes (both *P* < 0.01). Drugs including a P2Y12 receptor inhibitor of platelets (P2Y12 inhibitor), beta-blockers (BB), and statins were used more frequently in current smokers (*P* < 0.01, *P* < 0.01, *P* = 0.04, respectively). Freedom from all-cause death and CV death was lower in the non-smoker group (*P* < 0.001, *P* = 0.003, respectively). After adjustment, logistic regression revealed smoking was a major predictor for all-cause death and repeated PCI procedure [hazard ratio(HR): 1.71 and 1.46, respectively].

**Conclusions:**

Smoker's paradox extends to long-term outcome in patients with stable CAD undergoing PCI, which is partially explained by differences in baseline characteristics. However, smoking strongly predicted all-cause mortality and repeated PCI procedures in patients with stable CAD undergoing PCI.

## Introduction

Coronary artery disease (CAD) is a very common cardiovascular disease; except for standard coronary angiography, precise detection of CAD via deep learning technique and artificial intelligence are in development (1–7). Percutaneous coronary intervention (PCI) refers to coronary revascularization via various devices including balloon angioplasty or stent deployment. PCI is a common clinical practice in patients with stable CAD. Nevertheless, major adverse clinical events (MACE), including myocardial infarction (MI), revascularization and death can occur in patients after receiving PCI (8). Major risk factors, including diabetes mellitus (DM), hypertension dyslipidemia, and smoking could affect outcomes in patients with stable CAD who receive PCI. “Smoker's paradox” is not a new concept, it was firstly mentioned in 1995 to describe the unpredictable favorable outcome of reduced short-term mortality in smokers after acute coronary syndrome (9, 10). However, most studies describe smoker's paradox include both current and former smokers under the general classification of “smoking,” which might confound the true beneficial effect of nicotine withdraw; while excluding former smokers might clarify whether this phenomenon exist or not.

### Literature Review

The impact of smoking on outcomes in patients with acute coronary syndrome (ACS) or acute myocardial infarction (AMI) who receive PCI remains conflicting (11, 12). As for patients with ST elevation myocardial infarction (STEMI) receiving primary PCI, smokers had a similar one-year mortality rate compared with non-smokers (13, 14). On the contrary, some studies have revealed current smokers had better long-term outcomes compared with non-smokers with STEMI who were undergoing primary PCI. They also had more favorable post-infarction LV remodeling (15, 16). Given non-ST elevation acute coronary syndrome (NSTE-ACS), smokers seemed to have higher one-year mortality compared with non-smokers (17), while other study reported the existence of the smoker's paradox (18). The above differences were summarized in Table 1.

**Table 1:** Comparison of clinical cardiovascular outcomes among smokers and non-smokers.

**References**	**Pt number**	**Pt characteristics**	**Parameter**	**Results**
MC Cruz et al. (11)	2727	ACS	Current smokers Never smokers	Current smokers received more evidence-based treatments and had less in-hospital complications, in-hospital mortality and adverse outcomes at 1 year. More frequent percutaneous coronary intervention at 1 year was noted in current smoker
Weisz et al. (12)	2082	AMI	Former smokers Current smokers Never smokers	The “smoker's paradox” extends to patients undergoing primary PCI for AMI, with increased survival seen in current smokers
Redfors et al. (13)	2564	STEMI	Recent smokers Never smokers	In the present large-scale individual patient-data pooled analysis, recent smoking was unrelated to infarct size or microvascular obstruction, but was associated with a worse prognosis after primary PCI in STEMI.
Steele et al. (14)	1796	STEMI	Current smokers Ex-smokers Never-smokers	No evidence of an association between mortality and smoking status in patients with acute STEMI treated with PCI, and thus no evidence of a “smoker's paradox.”
Ciccarelli et al. (15)	713	STEMI	Current smokers Non-smokers	Not being a current smoker and ongoing DAPT at admission, in patients with STEMI undergoing PPCI, represent independent negative prognostic value.
Symons et al. (16)	471	STEMI	Smokers Non-smokers	Smoking is strongly and independently associated with intramyocardial hemorrhage at baseline. smoking was an independent predictor of more favorable post-infarction LV remodeling.
Robertson et al. (17)	13819	NSTE-ACS	Smokers Non-smokers	Smoking to be an independent predictor of higher 1-year mortality in patients presenting with NSTE-ACS and angiography study demonstrates CAD in smokers that is comparable to that in non-smokers but evident 1 decade earlier.
Amor-Salamanca et al. (18)	563	NSTE-ACS	Smokers Non-smokers	Confirms the “smoking paradox” amongst NSTACS patients, which is explained by the lower prevalence of previous myocardial infarction, diabetes or multivessel disease

*Pt number, patient number; Pt characteristic, patient characteristics; ACS, acute coronary syndrome; AMI, acute myocardial infarction; STEMI, ST-elevation myocardial infarction; NSTE-ACS, non-ST elevation acute coronary syndrome*.

### Hypothesis

In patients with stable CAD, current smokers have a greatly increased risk of future cardiovascular events, including mortality, compared with never-smokers (19). Other reported that after adjusting for differences in age, there did not appear to be any protective effect of smoking on 6-months cardiovascular outcomes in patients with stable CAD following PCI (20). Nevertheless, the effect of smoking on long-term outcomes in patients with stable CAD receiving PCI remains obscure. We hypothesize smoker's paradox extends to stable CAD patients undergoing PCI after long-term follow-up. Therefore, a longitudinal, prospective observational study was conducted to compare the influence of smoking on long-term outcomes between current smokers and non-smokers in patients with stable CAD after undergoing PCI. In addition, predictors for adverse clinical outcomes in both groups were further analyzed.

## Materials and Methods

### Study Design and Population

A prospective longitudinal study was conducted via catheterization data review from July 2011 through December 2018. Stable patients with CAD aged 20 to 90 years were recruited who underwent PCI at the inpatient clinic at the Taichung Tzu Chi Hospital, Taiwan. The patients were divided into two groups: current smokers or non-smokers. Patients with end-stage heart failure (HF), a previous history of malignancy, and scheduled PCI were excluded. Most patients were obtained during regular visits in the outpatient department (OPD). For patients who were lost to follow-up, a phone call was used to contact the patients themselves or their families. A survey of four-year clinical outcomes regarding all-cause death and major adverse cardiovascular events (MACE), including myocardial infarction, (MI), cardiovascular death (CV death), and repeated PCI procedures were completed at the end of the study. The Institution Review Board and ethics committee of Taichung Tzu Chi Hospital approved the study protocol, and no informed consent was required. This cohort study also fulfilled the guidance of Strengthening the Reporting of Observational Studies in Epidemiology (STROBE) statement (21).

### Data Processing, Measurements, and Analysis

Baseline characteristics, including body habitus, biochemical profiles, angiographic findings from cardiac catheterization, major risk factors, and variant therapeutic strategies such as drug medications and interventional procedures (balloon angioplasty, bare metal stent deployment, or drug-eluting stent deployment) were obtained. The criteria for major risk factors are described as follows: Diabetes Mellitus was defined as a fasting plasma glucose level > 126 mg/dL, a casual plasma glucose level > 200 mg/dL, or a hemoglobin A1c (HbA1c) level > 6.5% (22). Hypertension was defined as a BP of 140/90 mm Hg or higher, BP levels for which the benefits of pharmacologic treatment have been definitely established (23). Chronic kidney disease (CKD) was defined as an estimated glomerular filtration rate (eGFR) < 60 ml/min/1.73 m^2^, which is equal to or more than stage III chronic kidney disease (CKD) (24). Hypercholesterolemia was defined as a serum cholesterol level of more than 200 mg/dL or an LDL-C level > 100 mg/dL (25). Previous MI history was defined as a history of MI prior to index PCI, accompanied by a three-fold elevation of cardiac enzymes from the baseline value.

As for the hemodynamic data, central aortic pressures were measured continuously, and mean values were calculated during catheterization. Angiographic findings, including the number of diseased vessels and lesions were calculated, the lesion severity and complexity were evaluated via the synergy between PCI with Taxus Express paclitaxel-eluting stent (Boston Scientific, Marlborough, MA, USA) and cardiac surgery score (SYNTAX score) (26). Related clinical parameters including general characteristics, hemodynamic data, exposed risk factors, and interventional strategies were compared between current smokers and non-smokers. In addition, significant predictors for all-cause death and MACE were identified.

### Statistical Analysis

The analysis was primarily used to compare the differences between the two groups. Pearson's chi-squared test and Fisher's exact test was used to examine categorical variables. Analysis of variance (ANOVA) was used to test continuous variables. The log-rank test and Kaplan-Meier survival curves were used for comparing the survival difference. The Cox proportional hazards model was used to test the effect of independent variables on hazards. *P*-values < 0.05 were considered significant. All analyses were performed using SPSS for Windows, Version 24.0 (IBM Corp., Armonk, NY, USA).

## Results

### Baseline Characteristics of Study Population

During the study period, a total of 2,044 patients with stable CAD who had undergone PCI were assessed. Among them, 741 patients were current smokers and 1,303 patients were non-smokers, respectively. The mean follow-up time for current smokers and non-smokers was 49.3 ± 33.5 months and 48.0 ± 37.4 months, respectively (*P* = 0.43). Baseline clinical characteristics are listed in Table 2. Current smokers were younger than non-smokers (60.3 ± 12.4 vs. 65.9 ± 11.5 years old, *P* < 0.01). Current smokers had higher serum total cholesterol (179.8 ± 43.1 vs. 175.2 ± 44.1 mg/dL, *P* < 0.01) and low density lipoprotein cholesterol (LDL-C) levels (110.9 ± 37.9 vs. 105.9 ± 38.3 mg/dL). There was no difference in body mass index (BMI) between groups (*P* = 0.19). Given the hemodynamic data, current smokers had a lower central pulse pressure (CPP) than non-smokers (58.4 ± 20.3 vs. 63.9 ± 20.9 mmHg, *P* < 0.01).

**Table 2:** General characteristics of the study population among groups.

**Variable**	**Study groups**		***P*** **-value**
	**Current smoker** **(***N*** = 741)**	**Non-smoker** **(***N*** = 1303)**	
Age (years)	60.3 ± 12.4	65.9 ± 11.5	<0.01^*^
Weight (kg)	71.0 ± 12.4	66.3 ± 12.4	<0.01^*^
Height (meter)	1.66 ± 0.06	1.61 ± 0.09	<0.01^*^
BMI (kg/m^2^)	25.8 ± 3.9	25.5 ± 3.9	0.19
CSP (mmHg)	132.3 ± 23.9	135.8 ± 23.8	<0.01^*^
CDP (mmHg)	73.9 ± 13.4	71.9 ± 13.3	<0.01^*^
CPP (mmHg)	58.4 ± 20.3	63.9 ± 20.9	<0.01^*^
Cholesterol (mg/dl)	179.8 ± 43.1	175.2 ± 44.1	0.02
HDL (mg/dl)	38.0 ± 15.1	40.1 ± 16.5	<0.01^*^
TG (mg/dl)	154.9 ± 113.0	146.1 ± 94.1	0.07
LDL (mg/dl)	110.9 ± 37.9	105.9 ± 38.3	<0.01^*^
Creatinine (mg/dl)	1.6 ± 2.0	1.8 ± 2.1	0.12

*BMI, body mass index; CSP, central systolic pressure; CDP, central diastolic pressure; CPP, central pulse pressure; HDL, high-density lipoprotein cholesterol; LDL, low-density lipoprotein cholesterol; TG, triglyceride*.

* *significant*.

### Demographic and Clinical Data

Patient demographic and clinical data is shown in Table 3. Current smokers were mostly men (*P* < 0.01) and had a lower prevalence of DM and CKD, but a higher prevalence of previous MI compared with non-smokers (all *P* < 0.01). After the index PCI, current smokers were prescribed P2Y12 inhibitors, beta blockers, and statins more frequently than non-smokers (*P* < 0.01, *P* < 0.01, *P* = 0.04, respectively).

**Table 3:** Demographics and clinical data of study population, and medications prescribed after index PCI among groups.

**Variable**	**Study groups**		***P*** **-value**
	**Smoker** **(***N*** = 741)**	**Non-smoker** **(***N*** = 1,303)**	
Gender			<0.01^*^
Female	25 (3.4%)	483 (37.1%)	
Male	716 (96.6%)	820 (62.9%)	
Hypertension			0.74
No	331 (44.7%)	592 (45.4%)	
Yes	410 (55.3%)	711 (54.6%)	
DM			<0.01^*^
No	504 (68.0%)	735 (56.4%)	
Yes	237 (32.0%)	568 (43.6%)	
Previous MI			<0.01^*^
No	417 (56.3%)	862 (66.2%)	
Yes	324 (43.7%)	441 (33.8%)	
CKD			<0.01^*^
No	479 (64.6%)	706 (54.2%)	
Yes	262 (35.4%)	597 (45.8%)	
Stroke history			0.42
No	702 (94.7%)	1,223 (93.9%)	
Yes	39 (5.3%)	80 (6.1%)	
CABG history			0.12
No	739 (99.7%)	1,292 (99.2%)	
Yes	2 (0.3%)	11 (0.8%)	
Aspirin			0.37
No	66 (8.9%)	132 (10.1%)	
Yes	675 (91.1%)	1,171 (89.9%)	
P2Y12 inhibitors			<0.01^*^
No	90 (12.2%)	216 (16.6%)	
Yes	651 (87.8%)	1,087 (83.4%)	
Diuretics			0.23
No	602 (81.2%)	1,030 (79.0%)	
Yes	139 (18.8%)	273 (21.0%)	
BB			<0.01^*^
No	373 (50.3%)	735 (56.4%)	
Yes	368 (49.7%)	568 (43.6%)	
CCB			0.02
No	539 (72.7%)	885 (67.9%)	
Yes	202 (27.3%)	418 (32.1%)	
ACEI			0.31
No	594 (80.2%)	1,068 (82.0%)	
Yes	147 (19.8%)	235 (18.0%)	
ARB			0.30
No	535 (72.2%)	968 (74.3%)	
Yes	206 (27.8%)	335 (25.7%)	
Statin			0.04
No	426 (57.5%)	809 (62.1%)	
Yes	315 (42.5%)	494 (37.9%)	
Fibrate			0.07
No	692 (93.4%)	1,244 (95.5%)	
Yes	49 (6.6%)	59 (4.5%)	

*DM, diabetes Mellitus; CKD, chronic kidney disease alone; Previous MI, history of previous myocardial infarction; CABG history, history of coronary artery bypass graft; CKD, chronic kidney disease; P2Y12 inhibitor: P2Y12 receptor inhibitor of platelet. BB, beta-blockers; CCB, calcium channel blocker; ACEI, angiotensin-converting enzyme inhibitor; ARB, angiotensin receptor blocker*.

* *significant*.

**Figure 1:**
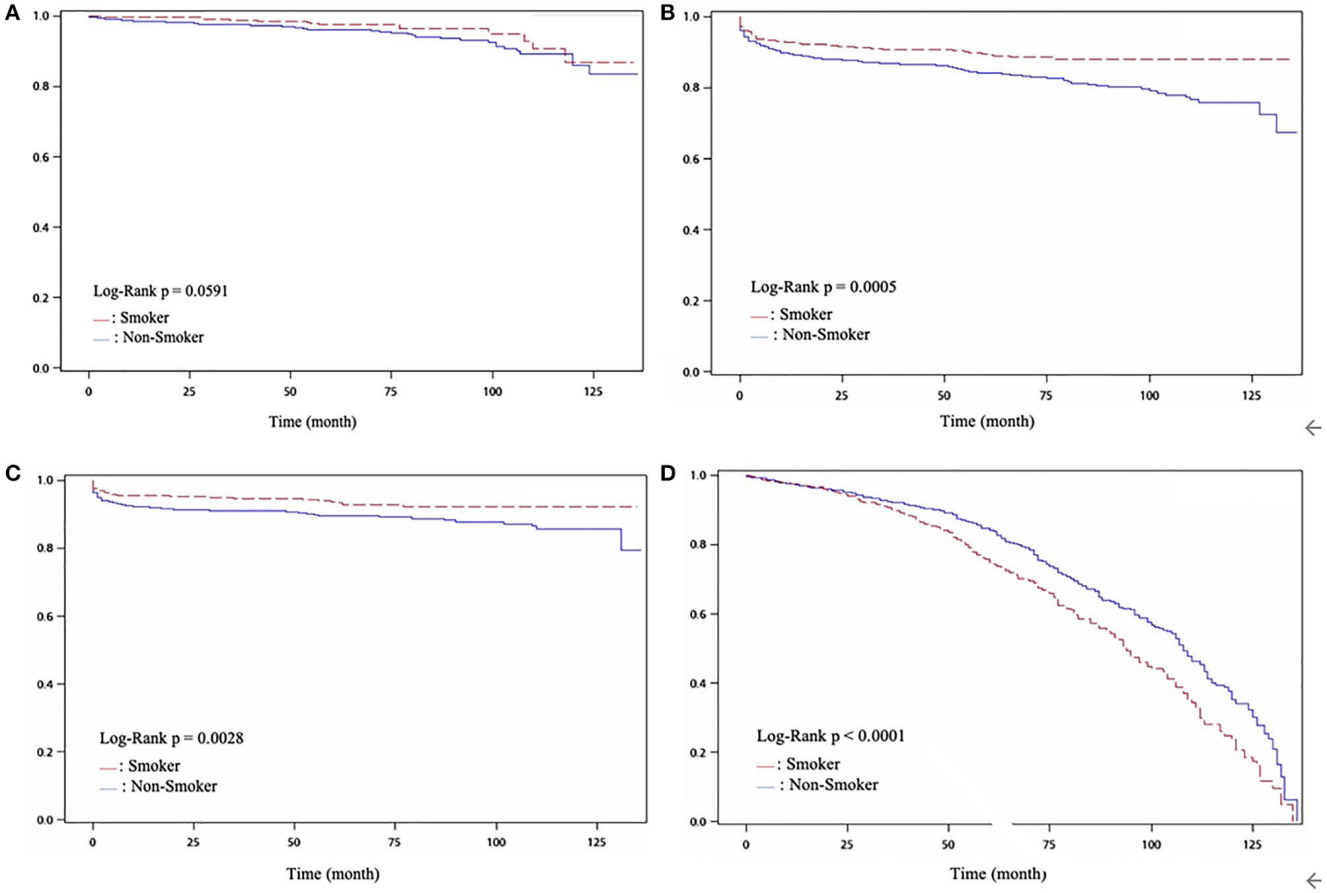
**(A)** Cumulative ratio of freedom from MI between two groups (*P* = 0.0591); **(B)** Cumulative ratio of freedom from all-cause death between two groups (*P* < 0.001); **(C)** Cumulative ratio of freedom from CV death between two groups (*P* < 0.003); **(D)** Cumulative ratio of freedom from Re-PCI between two groups (*P* < 0.001.

The results of angiographic findings and clinical outcomes are shown in Table 4. There was no difference in the number of diseased vessels, treated vessels, and treated lesions between both groups (all *P* = NS); however, current smokers had a higher SYNTAX score compared with non-smokers (*P* = 0.04). They also received more balloon angioplasty and bare-metal stent (BMS), but less drug-eluting stent (DES) deployment than non-smokers (*P* < 0.01). Current smokers had a lower rate of all-cause death and CV death than non-smokers (*P* < 0.01, *P* < 0.01, respectively); however, they had a higher rate of repeat PCI procedures than non-smokers (*P* < 0.01). The cumulative curve of freedom from MI, CV death, all-cause death, and repeated PCI procedures between the 2 groups is shown in Figure 1. Freedom from all-cause death and CV death was higher in the current smokers group (*P* < 0.001, *P* < 0.003, respectively), but they had a lower freedom from repeated PCI procedures (*P* < 0.001).

**Table 4:** Demography of angiographic findings and outcome among groups.

**Variable**	**Study groups**		***P*** **-value**
	**Smoker** **(***N*** = 741)**	**Non-smoker** **(***N*** = 1,303)**	
Follow-up time (months)	49.3 ± 33.5	48.0 ± 37.4	0.43
Number of diseased vessel			0.21
Single vessel disease	379 (51.2%)	717 (55.0%)	
Dual vessel disease	211 (28.5%)	331 (25.4%)	
Triple vessel disease	151 (20.4%)	255 (19.6%)	
Mean of treated vessels	1.2 ± 0.4	1.2 ± 0.4	0.35
Mean of treated lesions	1.4 ± 0.7	1.3 ± 0.7	0.23
SYNTAX score	11.5 ± 8.3	10.8 ± 7.9	0.04
LVEF	0.60 ± 0.10	0.60 ± 0.10	0.39
Type of intervention			<0.01^*^
Balloon angioplasty	144 (19.4%)	332 (25.5%)	
BMS deployment	306 (41.3%)	397 (30.5%)	
DES deployment	291 (39.3%)	574 (44.0%)	
MI			0.07
Yes	19 (2.6%)	52 (4.0%)	
No	722 (97.4%)	1251 (96.0%)	
CV death			<0.01^*^
Yes	44 (5.9%)	124 (9.5%)	
No	697 (94.1%)	1179 (90.5%)	
All-cause death			<0.01^*^
Yes	72 (9.7%)	195 (15.0%)	
No	669 (90.3%)	1108 (85.0%)	
Repeat PCI			<0.01^*^
Yes	232 (31.3%)	316 (24.3%)	
No	509 (68.7%)	987 (75.7%)	

*BMS, bare metal stent; DES, drug-eluting stent; LVEF, left ventricular ejection fraction; MI, myocardial infarction; Repeat PCI, repeated percutaneous coronary intervention; SYNTAX score, Synergy between Percutaneous Coronary Intervention with Taxus and Cardiac Surgery score*.

* *significant*.

### Predictors for Clinical Outcome

The predictors for all-cause death and MACE are shown in Table 5 (multivariate regression was used for this analysis). Before adjusting, age was related to all-cause death while smoking status was related to repeated PCI procedure. After adjusting, age no longer remained significant to any clinical outcome but smoking was found to be an independent risk factor for all-cause death and repeated PCI procedures after adjustment for age, DM, and CKD. As for predictors of clinical outcome, DM and previous MI history increased the risk of all-cause death, while usage of aspirin and angiotensin-converting enzyme inhibitor (ACEI) would reduce the risk of all-cause death. Use of aspirin and statins reduced the risk of CV death. Finally, CKD, high SYNTAX score, and use of P2Y12 inhibitors increased the risk of repeated PCI procedures; whereas usage of ACEI and statins would reduce the risk of repeated PCI procedures.

**Table 5:** Significant crude and adjusted predictors of outcome in Cox proportion hazard model for MI, All-death, CV-death, Repeated PCI.

**Variables**	**MI**		**All-death**		**CV-death**		**Repeated PCI**	
	**Crude HR (95%C.I.)**	**Adjusted HR (95%C.I.)^a^**	**Crude HR (95%C.I.)**	**Adjusted HR (95%C.I.)^a^**	**Crude HR (95%C.I.)**	**Adjusted HR (95%C.I.)^a^**	**Crude HR (95%C.I.)**	**Adjusted HR (95%C.I.)^a^**
Smoking	0.73 (0.40–1.33)	1.39 (0.53–3.68)	0.71 (0.48–1.03)	1.71 (1.11–2.62)^*^	0.79 (0.50–1.25)	1.58 (0.91–2.72)	1.54 (1.24–1.92)^**^	1.46 (1.17–1.83)^**^
Age	1.02 (0.99–1.05)	1.01 (0.96–1.06)	1.03 (1.02–1.05)^**^	1.00 (0.99–1.02)	1.03 (1.01–1.05)^**^	1.01 (0.99–1.04)	1.00 (0.99–1.01)	1.01 (1.00–1.02)
DM	1.49 (0.86–2.59)	1.14 (0.41–3.15)	1.31 (0.94–1.83)	1.46 (1.02–2.09)^*^	1.41 (0.93–2.16)	1.21 (0.74–1.98)	1.23 (0.99–1.54)	1.06 (0.85–1.33)
MI hx	2.77 (1.50–5.12)^**^	1.47 (0.42–5.17)	3.14 (2.18–4.52)^**^	1.68 (1.12–2.51)^*^	3.79 (2.35–6.11)^**^	1.51 (0.86–2.65)	1.26 (0.99–1.60)	1.22 (0.97–1.55)
CKD	1.29 (0.70–2.39)	1.27 (0.37–4.33)	2.39 (1.59–3.60)^**^	1.54 (0.97–2.47)	1.92 (1.15–3.19)^*^	1.66 (0.87–3.16)	1.50 (1.18–1.91)^**^	1.36 (1.07–1.74)^*^
Stroke	1.76 (0.69–4.48)	1.54 (0.40–5.97)	1.51 (0.88–2.59)	1.46 (0.81–2.63)	1.86 (1.01–3.43)^*^	1.33 (0.68–2.59)	0.94 (0.56–1.58)	1.32 (0.76–2.31)
CPP	1.00 (0.99–1.01)	1.00 (0.97–1.02)	1.00 (0.99–1.01)	0.99 (0.99–1.00)	1.00 (0.99–1.01)	0.99 (0.98–1.00)	1.00 (0.99–1.00)	0.99 (0.98–1.00)
Syntax core	1.04 (1.01–1.06)^*^	1.04 (0.99–1.09)	1.02 (1.01–1.04)^**^	1.00 (0.98–1.02)	1.03 (1.01–1.05)^*^	1.01 (0.99–1.03)	1.01 (0.99–1.02)	1.02 (1.01–1.04)^**^
DES	0.46 (0.22–0.96)^*^	1.30 (0.23–7.40)	0.83 (0.56–1.24)	0.76 (0.49–1.18)	0.60 (0.36–1.02)	1.23 (0.67–2.26)	0.99 (0.78–1.25)	1.09 (0.85–1.40)
Aspirin	1.62 (0.49–5.19)	0.65 (0.04–9.84)	1.16 (0.67–1.99)	0.55 (0.31–0.99)^*^	1.33 (0.64–2.78)	0.43 (0.19–0.99)^*^	1.59 (0.99–2.54)	0.72 (0.44–1.16)
P2Y12 inhibit	1.48 (0.67–3.28)	1.48 (0.30–7.29)	1.11 (0.69–1.79)	1.65 (0.97–2.82)	2.19 (1.03–4.64)^*^	2.31 (0.89–6.00)	1.59 (1.16–2.17)^**^	1.74 (1.25–2.42)^**^
BB	1.14 (0.66–1.98)	0.78 (0.27–2.27)	0.68 (0.48–0.96)^*^	0.94 (0.65–1.36)	0.61 (0.39–0.95)^*^	0.85 (0.51–1.44)	1.15 (0.93–1.41)	0.92 (0.75–1.16)
CCB	0.83 (0.42–1.64)	1.62 (0.47–5.63)	0.66 (0.43–1.00)	0.98 (0.63–1.51)	0.58 (0.33–1.03)	1.09 (0.57–2.08)	1.05 (0.83–1.33)	0.99 (0.78–1.27)
ACEI	1.19 (0.67–2.12)	0.51 (0.24–1.13)	0.72 (0.49–1.05)	0.52 (0.35–0.79)^**^	0.62 (0.38–1.01)	0.66 (0.37–1.19)	0.59 (0.46–0.75)^**^	0.44 (0.34–0.57)^**^
Statin	0.50 (0.27–0.93)^*^	0.54 (0.16–1.82)	0.37 (0.24–0.57)^**^	0.89 (0.55–1.43)	0.41 (0.24–0.70)^**^	0.53 (0.28–0.99)^*^	0.71 (0.57–0.88)^**^	0.68 (0.54–0.85)^**^

*DM, diabetes Mellitus; MI hx, Previous history of myocardial infarction; CKD, chronic kidney disease; CPP, central pulse pressure; SYNTAX score, Synergy between Percutaneous Coronary Intervention with Taxus and Cardiac Surgery score; DES, drug-eluting stent; P2Y12 inhibit, P2Y12 receptor inhibitor of platelet; BB, beta-blockers; CCB, calcium channel blocker; ACEI, angiotensin-converting enzyme inhibitor*.

a *Adjusted for MI, all-death, CV-death, and repeated PCI*.

* *P < 0.05*,

** *P < 0.01*.

## Discussion

The role of smoking on short-term outcomes in patients with ACS remains controversial. On the other hand, the influence of smoking on long-term outcomes in patients with stable CAD undergoing PCI is not clear. In contrast with adverse short-term cardiovascular outcomes observed in smokers, it seemed that current smokers have a better long-term prognosis in terms of all-cause mortality, cardiovascular mortality and repeated PCI procedures compared with non-smokers, and thus confirm our hypothesis. In addition, DM and CKD were predictors for all-cause death, while usage of aspirin and ACEI was associated with reduced risk. Finally, CKD, SYNTAX score, and use of P2Y12 inhibitors predicted repeat PCI procedures, but usage of ACE inhibitors and statins reduced the risk of repeat PCI.

In the present study, current smokers were 5 years younger than non-smokers, and there was no difference in the prevalence of hypertension between the two groups. However, central systolic pressure (CSP) was lower and central diastolic pressure (CDP) was higher in current-smokers compared with non-smokers, therefore current-smokers presented with a lower average central pulse pressure (CPP). This result might imply current smokers had less arterial stiffness and vascular resistance than non-smokers due to younger age, which is consistent with a previous study (27). On the other hand, although current smokers had a higher percentage of elevated serum total cholesterol and LDL-C, they also used statins more frequently than non-smokers after receiving PCI. This may negate the adverse effect of dyslipidemia on coronary atherosclerosis and clinical events.

Current smokers had a lower prevalence of DM and CKD, but a higher prevalence of MI history than non-smokers. This is compatible with a previous study that reported current-smokers had fewer adverse clinical profiles than non-smokers (28). Nevertheless, DM and MI history remained significantly correlated with all-cause death, while CKD was significantly related to repeated PCI procedures. This result is similar with previous studies that focused on adverse predictors for patients undergoing PCI. DM and previous MI have been reported to be predictors for all-cause death while CKD was a predictor for repeated PCI procedures (29).

Platelet P2Y12 inhibitors and statins were used more frequently in smokers compared with non-smokers after receiving PCI in the current study. Clopidogrel was used more often than ticagrelor and prasugrel was not available. The role of smoking on clopidogrel activity remains controversial. Some studies have concluded current smokers may have a higher clopidogrel metabolite exposure and pharmacodynamic effects than non-smokers (30), while cessation of smoking in clopidogrel-treated patients after PCI increased both VerifyNow P2Y12 platelet reaction units (PRUs) and platelet activity (31, 32). Another study postulated the lower PRUs were due to higher hemoglobin levels in current smokers than non-smokers, and there was no difference of PRUs after adjustment for hemoglobin (33). In our study, usage of P2Y12 inhibitors failed to reduce mortality but was associated with higher repeated PCI procedures. This was probably due to the high prevalence of previous MIs and multi-vessel disease in the study population. On the other hand, The use of ACEIs reduced all-cause death and repeat PCI procedures in patients with stable CAD irrespective of smoking status, which is consistent with previous reports (34, 35). Usage of statins also reduced CV death and the risk of repeated PCI procedures even though current-smokers and non-smokers had mildly elevated LDL-C levels. This suggests the use of statins could improve clinical outcomes in patients with stable CAD after receiving PCI even though there is a paucity of large scale clinical studies available.

Although current smokers had a lower rate of all-cause death and CV death than non-smokers, smoking was no more significant to both outcomes after adjusting for age, which implicates a lead-time bias might explain the smoking paradox in the survival analysis of all-cause death and CV death. There was no difference in the number or distribution of diseased vessels, treated vessels, and lesions between the two groups, but the SYNTAX score was higher in current smokers than non-smokers. Nevertheless, bare metal stents (BMS) deployment was performed more frequently in current-smokers; whereas, more drug-eluting stents (DES) were deployed in non-smokers. Collectively these factors might cause increased repeated PCI procedures in current smokers during long-term follow up (36).

Finally, we summarized the outcome differences between current smokers and non-smokers. In the long-term follow up, current smokers had a lower rate of all-cause death and CV death, but a higher rate of repeated PCI procedures than non-smokers. Based on the results of the adjusted regression model, we found smoking, DM and previous MI history predicted the hazard of all-cause death. Smoking, presence of CKD, SYNTAX score, and use of P2Y12 inhibitors predicted the hazard for repeated PCI. Aspirin could reduce both all-cause mortality and cardiovascular mortality. Although smoker's paradox still exists in patients with stable CAD undergoing PCI, it was explained by young age and few clinical adverse characteristics in current smokers. Smoking remained an adverse predictor for long-term clinical outcomes in patients with stable CAD undergoing PCI, this is firstly described. According to this study, cessation of smoking is recommended from viewpoint of preventive medicine, while aspirin usage could reduce long-term mortality in patients undergoing PCI whether smoke or not.

## Study Limitations

First, the amount of daily cigarette consumption and duration of smoking was not fully surveyed in this study, which might affect the extent of coronary atherosclerosis and thus influence the long-term outcome. Second, the age difference in study groups may cause lead-time bias, which could confound the survival analysis. Third, the number of MIs in both groups were too few to yield statistical significance, the possibility of inadequate follow-up time might exist. Fourth, despite of exclusion of end stage HF patients, a lower LVEF has an direct impact on mortality. This can be a potential confounding factor if the subgroup of patient with lower LVEF data were not captured in both groups. Finally, although clinical trials comparing the effect of smoking on stable CAD patients undergoing PCI is difficult for ethical reasons, whether aggressive cessation of smoking could improve long-term outcomes in smokers with stable CAD after receiving PCI remains to be clarified.

## Conclusions

The smoker's paradox extends to long-term outcome in stable CAD patients after undergoing PCI, which might be due to few clinical adverse characters in current smokers. However, smoking status independently predicted all-cause death and repeated PCI procedures in stable CAD patients after undergoing PCI. Further investigation of smoker's paradox between current smokers and ex-smokers via deep learning technique and artificial intelligence might be helpful.

## Data Availability Statement

The original contributions presented in the study are included in the article/supplementary materials, further inquiries can be directed to the corresponding author/s.

## Ethics Statement

The studies involving human participants were reviewed and approved by the Institution Review Board and Ethics Committee of Taichung Tzu Chi Hospital. Written informed consent was not required for this study, in accordance with the local legislation and institutional requirements.

## Author Contributions

M-JL and H-PW conceived and designed the study. S-LJ and S-LC collected the study data. C-CH performed the statistical analysis. H-PW and S-LJ drafted the manuscript. M-JL revised the manuscript. All authors had read and approved the final manuscript.

## Funding

This study was supported by a grant from the Department of Research, Taichung Tzu Chi Hospital, Taiwan and the China Medical University Hospital (Grant Number: C1090903010).

## Conflict of Interest

The authors declare that the research was conducted in the absence of any commercial or financial relationships that could be construed as a potential conflict of interest.

## Publisher's Note

All claims expressed in this article are solely those of the authors and do not necessarily represent those of their affiliated organizations, or those of the publisher, the editors and the reviewers. Any product that may be evaluated in this article, or claim that may be made by its manufacturer, is not guaranteed or endorsed by the publisher.
